# Revision of AUDIT Consumption Items to Improve the Screening of Youth Binge Drinking

**DOI:** 10.3389/fpsyg.2017.00910

**Published:** 2017-06-08

**Authors:** María-Teresa Cortés-Tomás, José-Antonio Giménez-Costa, Patricia Motos-Sellés, María-Dolores Sancerni-Beitia

**Affiliations:** ^1^Department of Basic Psychology, Faculty of Psychology, University of ValenciaValencia, Spain; ^2^Department of Methodology of the Behavioural Sciences, Faculty of Psychology, University of ValenciaValencia, Spain

**Keywords:** binge drinking, underage, AUDIT, alcohol screening, ROC

## Abstract

This study analyzes the appropriateness of an improved version of one of the most frequently used instruments for the screening of high-risk alcohol consumption. This adaptation was created in accordance with certain limitations recognized by other researchers and in an attempt to adjust the content and scales of some items to a more consensual definition of binge drinking. After revising items 2 and 3, the areas under the ROC curves of the AUDIT and of different abbreviated versions were calculated. A total of 906 minors (468 females) between the ages of 15 and 17 were evaluated. Stratified sampling was conducted on a population of high school students in the city of Valencia (Spain). One school was randomly chosen from each of the city’s 16 school districts. Information was collected on sociodemographic aspects, consumption patterns and the AUDIT containing the improved items. The percentage of underage BD reached 36%, regardless of gender or age. BD groups have been differentiated by different intensity levels, both in males and females. Upon comparing the effectiveness of the distinct versions of the AUDIT, it is recommended that researchers and clinics use the combination of the revised items 2 and 3 to ensure a more precise identification of underage BD. A cut-off point of 5 for this test would permit identification of 94% of the underage BD and would notably reduce false positives.

## Introduction

One of the most frequently used screening instruments for the identification of high-risk alcohol consumption in youth is the AUDIT and its abbreviated versions (Patton et al., 2014; Cortés et al., 2016; Hagman, 2016) which was designed to identify persons with hazardous and harmful patterns of alcohol consumption (Babor et al., 2001). Specifically, research on the young brain refers mainly to these tools to compile consumption data and classify youth as either binge drinking (BD) or no binge drinking (non-BD) (Mota et al., 2013; López-Caneda et al., 2014a,b). Other studies have used the AUDIT score for correlation with structural and functional aspects of certain brain areas (Wahlstrom et al., 2012; Howell et al., 2013; Smith and Mattick, 2013; Kvamme et al., 2015).

Of the three dimensions included in the AUDIT (quantity-frequency, symptoms of dependency, and consequences of consumption), the first of these dimensions is the most frequently used to determine consumption in youth (Chung et al., 2002; Thomas and McCambridge, 2008; Seguel et al., 2013). The three items making up this first dimension, AUDIT-C, obtain higher sensitivity and specificity values in the detection of high-risk consumption as compared to the overall scale (DeMartini and Carey, 2012; Barry et al., 2015; Cortés et al., 2016; García et al., 2016).

These results support the conclusions obtained in the revision conducted by Clark and Moss (2010) with regards to the abbreviated AUDIT versions appearing to be more useful for youth, even when limited to item 3. This item, used to classify underage BD, has revealed psychometric properties that are similar to those of the AUDIT-C (Bowring et al., 2013; Blank et al., 2015; Paiva et al., 2015).

Despite the fact that they are very frequently used instruments, limitations have been suggested with regards to their efficiency in identifying BD. On the one hand, reference has been made to the measurement scales used for the different items. Letourneau et al. (2017) warned that in item 3, a drinker who engaged in three BD days per week (e.g., Friday through Sunday) is forced to describe their drinking as either “*weekly”* or “*daily or almost daily”* on the AUDIT-C, even though said drinking took place only three times a week. For Question 2, the numerical amount for any respondent who reports consuming 10 or more drinks on a typical day, whether it is 12, 15, or 30 drinks, will be coded as 10.

On the other hand, in an attempt to better identify underage BD, an effort has been made to more precisely specify the cut-off points of the scales. In this regard, no consensus has been reached either, and there is still a very wide range for the AUDIT, varying between 2 and 10 points (Knight et al., 2003; Kelly et al., 2004; Clark and Moss, 2010). For minors, the most frequently used cut-off point is 4 (Chung et al., 2002; Santis et al., 2009; Cortés et al., 2016) and 3 in the AUDIT-C (Chung et al., 2002; Cortés et al., 2016).

Furthermore, some researchers have tried out new combinations of items in order to better predict the pattern of underage consumption. Again, in this case, consensus has yet to be reached. McCambridge and Thomas (2009) allude to the fact that the best combination would consist of items 3, 5, and 8. Bowring et al. (2013) suggest that the best combination is 3, 4, 8, and 9. More recently, Blank et al. (2015) referred to separately using items 2 and 3, increasing the number of response options to obtain more precise information on the consumption pattern. In this way, sensitivity and specificity of the items are improved until reaching 0.8 and 0.7, respectively. Furthermore, some studies have noted the low correlation of item 1 with the total of the scale (Gmel et al., 2001; McCambridge and Thomas, 2009), recommending its elimination.

All of this disagreement has led to an interest in making improvements in the wording of the consumption items (AUDIT-C) given that these are the most explanatory of the youth consumption pattern. Included in the suggested changes is the modification of item 3, reducing the number of drinks (five or more on one consumption occasion -Kokotailo et al., 2004-; four or more drinks for women and five or more drinks for men -Olthuis et al., 2011-); or transforming the number of drinks to standard drinking units (SDUs), according to the country of origin (García et al., 2016). Other proposals have narrowed the time limit to “one single consumption occasion” in item 2 (García et al., 2016), although it has also been suggested that grams of alcohol should be used instead of number of drinks to evaluate the quantity ingested for this item (Gmel et al., 2001).

None of the suggested improvements has been overwhelmingly accepted by researchers, perhaps because they do not comply with a consensual definition of BD. Recent revisions of the operationalization of this consumption pattern (Courtney and Polich, 2009; Parada et al., 2011; Cortés and Motos, 2016) coincide in identifying the National Institute on Alcohol Abuse, and Alcoholism [NIAAA] (2004) definition as being the most well-adjusted, although limiting it to consumption engaged in over the past 6 months – given that it is intermittent behavior- and adapting it to the SDU value of each country. In the case of Spain, BD is identified as the consumption, during a 2 h interval, of six or more SDUs for women and seven or more for men, at least once over the past 6 months. Furthermore, it is important to note that this definition only establishes a limit for a very heterogeneous group of consumers; therefore it is necessary to differentiate the most homogenous subgroups possible.

In this work, we have modified the content of the consumption items included in the AUDIT-C, adapting them both in terms of wording as well as in their measurement scales, to the proposed consensual definition of BD. This shall permit the identification of which of these items best classifies heavy youth drinkers, and therefore, shall optimize the selection of BD sample participants, thereby improving the precision of the obtained results.

## Materials and Methods

### Participants

Nine hundred and six participants, 468 women and 438 men, took part in the study. Their ages ranged from 15 to 17, with mean age *M* = 15.99 years, *SD* = 0.8 years. All of the participants were high school students. **Table 1** shows the distribution of the participants based on gender, age and whether or not they engage in BD. Overall, 36.1% of these adolescents (*n* = 327) engaged in BD, 52.9% (*n* = 173) were female and 47.1% (*n* = 154) were male. Differences were not found based on gender [*F*(1,904) = 0.191; *p* = 0.612], or age [*F*(1,904) = 3.929; *p* = 0.54].

**Table 1:** Demographic characteristics of sample.

	BD (*N* = 327 // 36.1%)			Non-BD (*N* = 579//63.9%)		
	Male *N* (%)	Female *N* (%)	Total *N* (%) [% of BD]	Male *N* (%)	Female *N* (%)	Total *N* (%) [% of Non-BD]
15 years old	42 (47.7%)	46 (52.3%)	88 (100%) [26.9%]	86 (44.3%)	108 (55.7%)	194 (100%) [33.5%]
16 years old	61 (45.9%)	72 (54.1%)	133 (100%) [40.7%]	112 (50.7%)	109 (49.3%)	221 (100%) [38.2%]
17 years old	51 (48.1%)	55 (51.9%)	106 (100%) [32.4%]	86 (52.4%)	78 (47.6%)	164 (100%) [28.3%]
Total *N* (%)	154 (47.1%)	173 (52.9%)	327 (100%)	284 (49.1%)	295 (50.9%)	579 (100%)

*BD, binge drinkers*.

### Procedure

Stratified sampling was carried out on a population of mandatory secondary school (grades 7–10), upper secondary (grades 11–12), and vocational training students in the city of Valencia (Spain). One school was randomly chosen from each of the 16 school districts in the city. Questionnaires were administered in classrooms during the school day. In all cases, participation was voluntary and anonymous.

A self-report diary was used, in which, for each day of the week, participants were to indicate the type and number of drinks consumed and the approximate time when the drinking took place. Each use was converted to grams of alcohol, based on the Spanish SDU (1 hard liquor = 20 g; 1 beer/wine = 10 g) (Rodríguez-Martos et al., 1999). This value was multiplied by the number of glasses of each type of alcoholic beverage that were consumed.

Based on the SDUs consumed and the number of hours in which this consumption took place, participants were classified as BD or non-BD. In all cases, there was compliance with the consumption proportion of seven or more SDUs in a 2 h interval for males and the consumption of six or more SDUs during the same time interval for females (National Institute on Alcohol Abuse, and Alcoholism [NIAAA], 2004).

Participants also filled out the 10 AUDIT items (Spanish version validated by Contel Guillamon et al., 1999). Three variables were extracted from this instrument: the sum of the 10 items (AUDIT), the sum of the first three items (AUDIT-C), and the score on the third question (AUDIT-3). In this study, the internal consistency of the AUDIT and the AUDIT-C was 0.74 and 0.83, respectively.

Next, the consensual definition of BD was used to improve item 3. It was worded as follows: *During the past 6 months, what is the average number of days per month with BD consumptions (seven or more Spanish SDUs for males and six or more SDUs for females over a 2 h period*)? The response scale was adapted based on the results obtained in prior studies conducted with minors and university students (Patrick et al., 2013; Cortés et al., 2016; Hagman, 2016). Following the revision of consumption quantity and frequency, it is considered more representative to use response alternatives that qualify normal situations, such as that some youth have engaged in BD once over the past 6 months, hence alternative 1 which considers this behavior to be sporadic and different from that of the other alternatives. The measurement scale definitively consists of the following: *(0) Never; (1) Sporadically -less than once a month-; (2) between 1 and 4 times; (3) between 5 and 8 times; (4) between 9 and 12 times; (5) 13 or more times.*

The wording of item 2 was also improved, changing number of drinks for number of Spanish SDUs consumed in 1 day. Finally, it is worded as follows: *How many SDUs do you tend to have on a day when you drink alcohol?* And maintaining its original response scale *(0) 1 or 2; (1) 3 or 4; (2) 5 or 6; (3) 7 to 9; and (4) 10 or more.*

Then, based on self-reports, these two new variables were generated. Later the value of the AUDIT-CR was calculated (A1+A2revised+A3revised), and the usefulness of the A3revised item was assessed. Finally, considering the recommendations from some prior studies, the A2revised+A3revised variable was also calculated.

### Statistical Analyses

Four cluster analyses were also conducted with the BD and non-BD youth, based on the values of *number of grams consumed in a BD session* and *number of hours of consumption* for females and for males. In all cases, the extraction procedure consisted of two phases, which led to a natural classification of the subjects into different groups.

An analysis of variance (ANOVA) was performed, with its corresponding *a posteriori* tests, using the eight groups obtained in the clusters as independent variables (IVs) to determine whether there were differences in the grams consumed and the number of hours.

The area under the ROC (*Receiver Operating Characteristic*) curve was calculated using the method proposed by Hanley and McNeil (1983), which provides a graphic representation of a classifier’s performance.

To determine the optimal AUDIT cut-off score, our goal was to minimize false negatives and thus improve, as much as possible, the detection of youth engaging in this activity. Therefore, cut-off scores that maximized sensitivity were used. This methodology is based on prior studies (Cortés et al., 2016; Cortés Tomás et al., 2017). In the absence of a gold standard, Zweig and Campbell (1993) suggest using a consensus or majority expert opinion. As described in the introduction, the gold standard used in this study was consumption during a 2 h interval of ≥6 SDUs for women and ≥7 SDUs for men at least once over the past 6 months.

It is possible to compare the discriminatory capacity of the different versions of this screening tool based on their respective ROC curves, given that they were measured simultaneously, were applied to the same subjects and were contrasted with the same consensual definition of the revisions of BD operationalization.

## Results

The cluster analysis among BD females produced two differentiated groups (BD1F/BD2F) (**Table 2**). In the case of the BD males, two groups were produced (BD1M/BD2M). Of the non-BD, two female (NONBD1F/NONBD2F) and two male (NONBD1M/NONBD2M) groups were produced.

**Table 2:** Binge drinking (BD) and non-binge drinking (non-BD) groups differentiated by sex resulting from the clusters analyses.

	Cluster	*n* (%)	Mean grams (*SD*)	Mean consumption hours (*SD*)
**BD**				
Female	BD1F	52 (30.0)	186.1 (65.3)	4.94 (1.6)
	BD2F	121 (70.0)	82.97 (24.1)	2.14 (0.8)
				
Male	BD1M	104 (67.5)	97.98 (22.9)	2.29 (0.8)
	BD2M	50 (32.5)	212.6 (71.3)	4.68 (1.3)
**Non-BD**				
Female	NONBD1F	233 (79.0)	37.45 (15.6)	1.83 (0.9)
	NONBD2F	62 (21.0)	91.92 (31.6)	4.90 (1.4)
				
Male	NONBD1M	213 (75.0)	40.43 (18.3)	1.92 (0.97)
	NONBD2M	71 (25.0)	115 (45.2)	5.8 (1.96)

*BD, binge drinkers; SD, standard deviation; BD1F, group one females of binge drinkers; BD2F, group two of females binge drinkers; BD1M, group one of males binge drinkers; BD2M, group two of males binge drinkers; NONBD1F, group one of NON females binge drinkers; NONBD2F, group two of NON females binge drinkers; NONBD1M, group one of NON mles binge drinkers; NONBD2M, group two of NON males binge drinkers*.

The ANOVA performed among the eight groups (four BD and four non-BD) indicated that there were significant differences in the number of grams consumed [*F*(7,898) = 326.905; *p* < 0.0001] and in the number of consumption hours [*F*(7,898) = 203.304; *p* < 0.0001].

Upon comparison of the four BD groups (**Table 3**), it was found that the subgroups consuming the larger number of grams (BD1F and BD2M) took twice the amount of time in drinking this quantity. Furthermore, both are similar in terms of quantity consumed, as well as in time spent drinking.

**Table 3:** A posteriori Games-Howell test.

(I) Clusters_only_BD	(J) Clusters_only_BD	Difference in means (I–J)	Std. error	Significant	95% confidence interval (lower bound – upper bound)
**Alcohol grams**						
BD1F	BD2F	103.187(ˆ*)	9.322	0.000	73.86	132.52
	BD1M	88.173(ˆ*)	9.336	0.000	58.81	117.54
	BD2M	-26.446	13.562	0.521	-68.45	15.56
BD2F	BD1M	-15.014(ˆ*)	3.133	0.000	-24.60	-5.43
	BD2M	-129.633(ˆ*)	10.325	0.000	-162.19	-97.07
BD1M	BD2M	-114.619(ˆ*)	10.337	0.000	-147.21	-82.03

**Hours**						
BD1F	BD2F	2.802(ˆ*)	0.238	0.000	2.05	3.55
	BD1M	2.654(ˆ*)	0.240	0.000	1.90	3.41
	BD2M	0.262	0.295	0.986	-0.65	1.18
BD2F	BD1M	-0.148	0.105	0.853	-0.47	0.17
	BD2M	-2.540(ˆ*)	0.201	0.000	-3.17	-1.91
BD1M	BD2M	-2.392(ˆ*)	0.203	0.000	-3.03	-1.75

*(^∗^) The difference in means is significant at the 0.05 level*.

*BD, binge drinking; Std. error, standard error; BD1F, group one of females binge drinkers; BD2F, group two of females binge drinkers; BD1M, group one of males binge drinker; BD2M, group two of males binge drinkers*.

Of the non-BD females, it is noteworthy that the NONBD2F group consumes a similar quantity of grams as the BD2F and BD1M groups, but it does so over a much longer time period, equivalent to that of groups BD1F and BD2M.

As for the non-BD males, the NONBD2M group is similar to BD1F in terms of quantity of grams consumed but it takes a greater number of hours to do so, therefore this is not considered BD.

When considering all of the interviewees, differentiated according to the eight resulting groups of the BD/non-BD clusters, the three classic versions of the AUDIT yielded lower values in the area under the ROC curve as compared to the results obtained for the modified versions of this instrument (**Table 4**). This area ranges from 0.741 in the case of the AUDIT to 0.801 in the case of the AUDIT-C.

**Table 4:** Performance of the three versions of the AUDIT in detecting binge drinking for the entire sample.

	Cut-off	Sensitivity	Specificity	ROC (95% confidence interval)
AUDIT	≥5	1.000	0.264	0.741 (0.681–0.801)
	≥6	1.000	0.316	
	≥7	0.940	0.383	
	≥8	0.860	0.455	
	≥9	0.820	0.535	
	≥10	0.740	0.617	
	≥11	0.580	0.690	

AUDIT-C	≥4	1.000	0.305	0.801 (0.751–0.852)
	≥5	1.000	0.339	
	≥6	0.960	0.420	
	≥7	0.920	0.525	
	≥8	0.780	0.671	
	≥9	0.600	0.792	

AUDIT-3	≥1	0.980	0.336	0.752 (0.696–0.808)
	≥2	0.780	0.610	

AUDIT-CR	≥5	1.000	0.527	0.888 (0.856–0.920)
	≥6	0.960	0.655	
	≥7	0.920	0.741	
	≥8	0.700	0.853	

A2R+A3R	≥4	1.000	0.697	0.898 (0.871–0.925)
	≥5	0.940	0.746	
	≥6	0.700	0.872	
	≥7	0.140	0.989	

AUDIT-3R	≥1	0.980	0.688	0.883 (0.854–0.913)
	≥2	0.700	0.850	

*ROC, receiver operating characteristic*.

The adjustment of the AUDIT questions to the definition of what is considered BD allows for the significant increase in the area under the ROC curve. Both when considering the AUDIT-CR, which includes the revision of the two items as well as when considering the A3R, the ROC area reaches 0.88.

**FIGURE 1:**
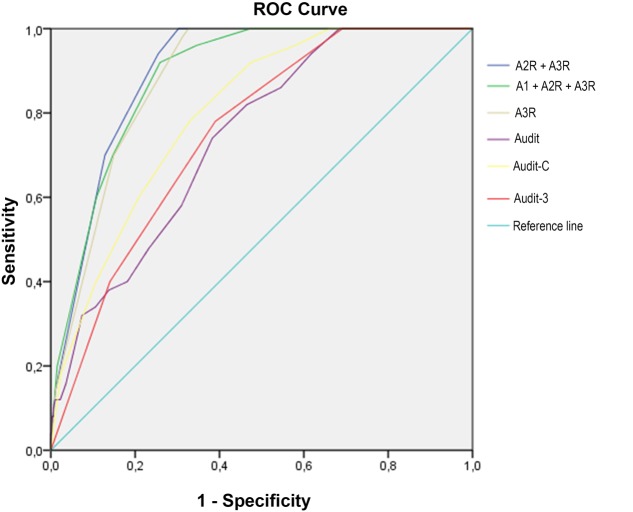
Receiver operating characteristic (ROC) curves for all AUDIT versions to detect BD.

But the most parsimonious combination that also permits a slight increase in the explained area is the one that includes the sum of the A2R and A3R (**Figure 1**).

Using the score of 5 on the A2R+A3R, 94% of the BD young people were detected (sensitivity) and 75% of the non-BD (specificity). When the cut-off score was established at 4, the sensitivity increased slightly, but the specificity was much worse.

## Discussion

This study analyzes the appropriateness of an improved version of the AUDIT. The adaptation has been carried out based on the limitations recognized by other researchers (McCambridge and Thomas, 2009; Olthuis et al., 2011; Bowring et al., 2013; Blank et al., 2015; Cortés et al., 2016; García et al., 2016; Letourneau et al., 2017) and by attempting to adjust the content and the scales of some items to a more consensual definition of BD.

Within the group of heavy drinkers, the underage population warrants special attention due to the potential repercussions on its bio-psycho-social development and maturity (Squeglia et al., 2011; Pascual et al., 2014). In Spain, 4 out of every 10 minors have access to this substance which is not legally authorized until the age of 18, eventually engaging in BD (Observatorio Español sobre Drogas [OED], 2016). This same percentage has been observed in the population of youth evaluated in this study.

Furthermore, the presence of females of this age is also evident, confirming the trend that has been warned of in prior national epidemiological surveys (Observatorio Español sobre Drogas [OED], 2016) that found a similar number of males and females engaging in intense alcohol consumption.

Our findings provide further insight into the understanding of the existence of different subgroups within the BD collective, both males and females, based on the seriousness of their behavior -a greater quantity of alcohol consumed, for more hours and at a greater frequency-. In addition, it should not be forgotten that among the BD groups that consume the most, both males and females drink similar amounts of alcohol and they do so in the same number of hours. This leads to a clearly greater risk for females, given that they are more vulnerable to the consequences of alcohol consumption. Furthermore, this result quantifies results of previous research (Valencia-Martín et al., 2007; Pilatti et al., 2013) claiming that there is a higher level of consumption by BD males, limiting it only to the subgroups that consume less.

The healthcare and social implications that are generated in the BD minors would be reduced if it were possible to detect and intervene in this behavior as early as possible. This suggests the need for sufficiently powerful screening measures to identify this consumption pattern with the least possible error. This would offer improvements not only in the clinical and prevention areas but also in the area of research (Foxcroft et al., 2015; Walton et al., 2015; Arnaud et al., 2016) given that a more adjusted classification of the subjects would permit greater precision in the obtained results.

As found in the literature that was consulted (DeMartini and Carey, 2012; Barry et al., 2015; Cortés et al., 2016; García et al., 2016), of all of the AUDIT versions used, the AUDIT-C is the version that classifies adolescents by improving the correct identification of the non-BD, compared to the AUDIT. However, upon transforming the items, adjusting them through both wording and in response scale to the most widely accepted BD definition, the adjustment of identification of this consumption pattern is increased.

Upon comparing the three versions of the revised AUDIT -AUDITCR/A3r/A2r+A3r- the last combination stands out (A2r+A3r) given that it identifies the greatest number of BDs and reduces the number of false positives. It may be stated that the recommendations of Blank et al. (2015) to focus on items 2 and 3, as well as those of Gmel et al. (2001) and McCambridge and Thomas (2009) to ignore item 1, contribute to an improved classification of BD. In addition to this, if we add improvement in the wording of the items and their response scales, adjusting them to the operational definition of BD, a greater area is obtained under the ROC curve. This suggests that this is a test with the greatest discriminatory capacity of all evaluated in this study. Having an instrument with an area under the ROC curve of 0.898 means that there is an 89.8% probability that, when considering two randomly selected minors, one BD and the other non-BD, the test will correctly classify them.

The reliability obtained through this new combination of items is very similar to that of the complete original scale -0.74, qualified as an acceptable reliability coefficient-. This result is not surprising, given that the items have been reformulated in order to note different aspects of BD. Item 2 reveals a more than intense consumption, as it is conducted over one entire day, whereas item 3 notes the frequency with which BD is engaged in. The combination of both not only informs of having reached a limit in BD in the form of overconsumption, but also if the youth drinks in a manner that extends over a longer period of time.

## Conclusion

Despite the fact that the AUDIT and its abbreviated versions appear to be appropriate tools to screen adolescents who are engaging in this behavior, the identification of heavy drinkers is improved by using a more parsimonious combination of two items. Even in those cases in which researchers recur to item 3 in order to classify BD/non-BD (Bowring et al., 2013; Mota et al., 2013; López-Caneda et al., 2014b; Blank et al., 2015) it would be more appropriate, given the notable improvement in discrimination of this test, to recur to the revised item 3.

In fact, it is recommended that researchers and clinics use the combination of the two items (A2r+A3r) proposed in this work for a more precise identification of BD minors. Specifically, starting from a cut-off point of 5, it may be possible to identify 94% of the underage BD. The sensitivity and specificity values attained are three points higher than those achieved using the three-item combination proposed by McCambridge and Thomas (2009), but using one less item, facilitating its applicability.

Our study may be limited in that it relies on self-reporting. This method of data collection has been questioned in adult samples, given that it may present an underestimation of consumption (Smith et al., 1990). However, in adolescent populations, self-reports have been found to be reliable and valid when conducted in a confidential manner, compared with other survey protocols (e.g., household survey) (Winters et al., 1990; Knight et al., 2003) in which youth perceived they were at great risk of being identified (Fowler and Stingfellow, 2001; Degenhardt et al., 2013).

According to the recommendations made by Santis et al. (2009), additional research is necessary in order to generalize these results to other geographic areas.

## Ethics Statement

It was not necessary for the study because there was no ethics relevant problems. People just filled out questionnaires/tests, afterward they got feedback on their scores. No manipulation or violation was done. The study was undertaken in compliance with Spanish legislation (approved by the Department of Education) and the code of ethics for research involving human subjects outlined by the University of Valencia Human Research Ethics Committee. The adolescents and their legal representatives signed an informed consent form.

## Author Contributions

M-TC-T and J-AG-C conceived of the study and collected the data. M-TC-T and M-DS-B analyzed the data. M-TC-T, PM-S, and J-AG-C wrote the paper. M-TC-T, J-AG-C, PM-S, and M-DS-B approved the final version to be published.

## Conflict of Interest Statement

The authors declare that the research was conducted in the absence of any commercial or financial relationships that could be construed as a potential conflict of interest.
